# Genetic analysis of phenylpropanoids and antioxidant capacity in strawberry fruit reveals mQTL hotspots and candidate genes

**DOI:** 10.1038/s41598-020-76946-x

**Published:** 2020-11-19

**Authors:** Delphine M. Pott, José G. Vallarino, Eduardo Cruz-Rus, Lothar Willmitzer, José F. Sánchez-Sevilla, Iraida Amaya, Sonia Osorio

**Affiliations:** 1 Departmento de Biología Molecular y Bioquímica, Instituto de Hortofruticultura Subtropical y Mediterránea “La Mayora”, Universidad de Málaga-Consejo Superior de Investigaciones Científicas, Campus de Teatinos, 29071 Málaga, Spain; 2 Unidad Asociada de I + D + i IFAPA-CSIC Biotecnología y Mejora en Fresa, Málaga, Spain; 3 Laboratorio de Genómica y Biotecnología, Instituto Andaluz de Investigación y Formación Agraria y Pesquera (IFAPA), Centro IFAPA de Málaga, 29140 Málaga, Spain; 4 Max-Planck-Institut Für Molekulare Pflanzenphysiologie, Potsdam-Golm, Germany

**Keywords:** Natural variation in plants, Plant breeding, Plant genetics, Secondary metabolism

## Abstract

Phenylpropanoids are a large class of plant secondary metabolites, which play essential roles in human health mainly associated with their antioxidant activity. Strawberry (*Fragaria* × *ananassa*) is a rich source of phytonutrients, including phenylpropanoids, which have been shown to have beneficial effects on human health. In this study, using the *F.* × *ananassa* ‘232’ × ‘1392’ F_1_ segregating population, we analyzed the genetic control of individual phenylpropanoid metabolites, total polyphenol content (TPC) and antioxidant capacity (TEAC) in strawberry fruit over two seasons. We have identified a total of 7, 9, and 309 quantitative trait loci (QTL) for TPC, TEAC and for 77 polar secondary metabolites, respectively. Hotspots of stable QTL for health-related antioxidant compounds were detected on linkage groups LG IV-3, LG V-2 and V-4, and LG VI-1 and VI-2, where associated markers represent useful targets for marker-assisted selection of new varieties with increased levels of antioxidant secondary compounds. Moreover, differential expression of candidate genes for major and stable mQTLs was studied in fruits of contrasting lines in important flavonoids. Our results indicate that higher expression of *FaF3′H*, which encodes the flavonoid 3′-hydroxylase, is associated with increased content of these important flavonoids.

## Introduction

**Figure 1:**
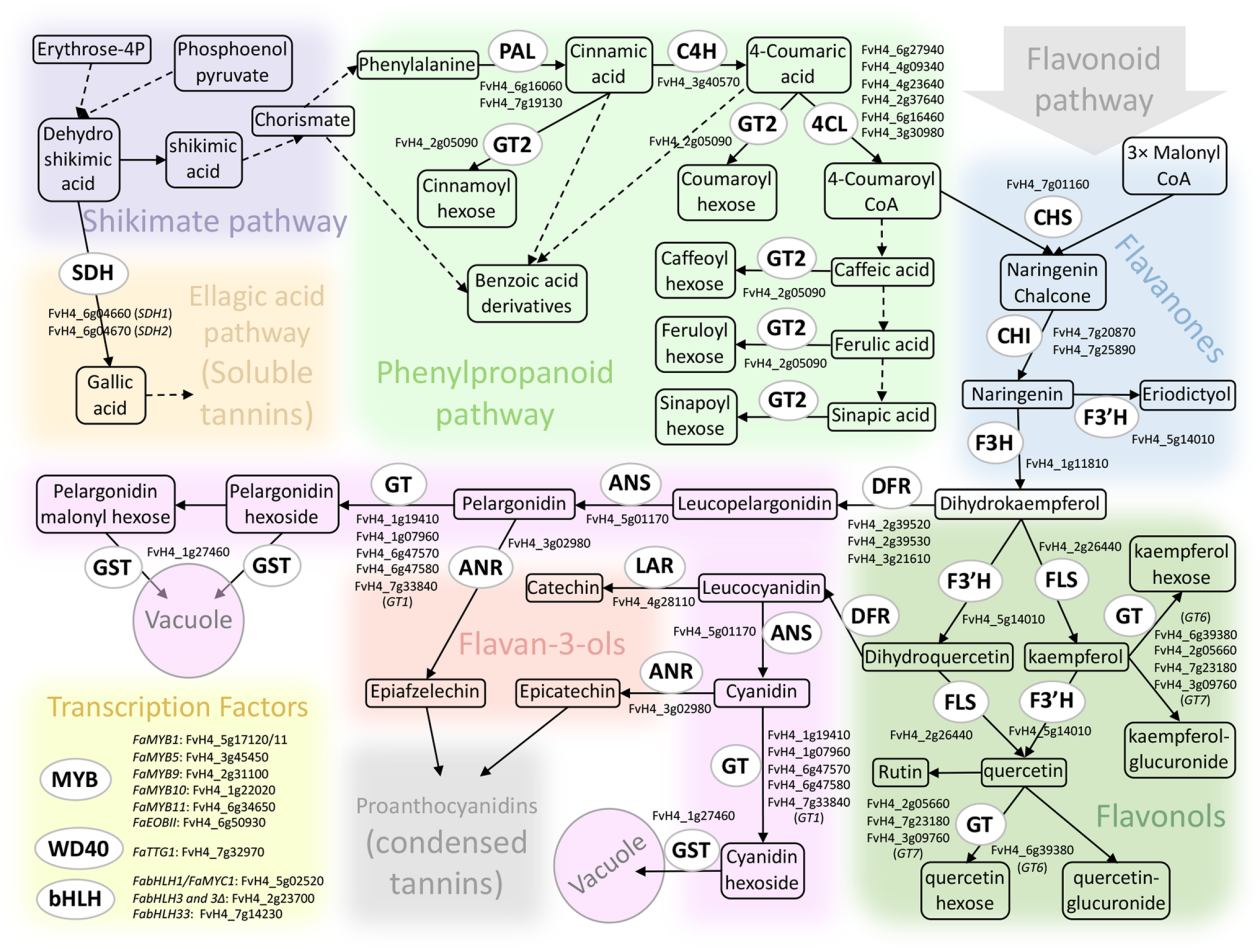
Schematic representation of shikimate, phenylpropanoid and flavonoid biosynthesis in strawberry. Adapted from Fragariacyc (https://www.rosaceae.org/), and^34,58,59^. Characterized enzymes and transcription factors regulating biosynthetic genes are shown, as well as gene numbers according to the *F. vesca* reference genome v.4. ANS, anthocyanidin synthase; ANR, anthocyanidin reductase; C4H, cinnamic acid 4-hydroxylase; CHI, chalcone isomerase; CHS, chalcone synthase; 4CL, 4-coumaroyl-CoA ligase; DFR, dihydroflavonol reductase; F3H, flavanone 3-hydroxylase; F3′H, flavonoid 3′-hydroxylase; FLS, flavonol synthase; GT1, anthocyanidin glucosyltransferase; GT2, (hydroxy)cinnamic acid and (hydroxy)benzoic acid glucosyltransferase; LAR, leucoanthocyanidin reductase; PAL, phenylalanine ammonia lyase; SDH, shikimate dehydrogenase.

Fruit and vegetable are a major component of the human diet, promoting healthy ageing by reducing risks of a wide array of chronic and degenerative diseases^1–4^. Strawberry is a crop of particular interest for a healthy diet as the fruit is a rich source of nutrients with high antioxidant activity^5^. Strawberry fruit is particularly rich in flavonoids, a subgroup of polyphenols, which are one of the most extensive and heterogenous family of secondary metabolites^6–8^. Flavonoids are involved in several important physiological processes, such as regulation of auxin transport, male fertility, biotic and abiotic stress responses, and fruit and flower pigmentation^9–12^. In general, flavonoids are subclassified into different families that comprise 6 subclasses according to their structure: flavonols, flavones, flavanone, flavanols or flavan-3-ols (flavan-3-ol monomers, proanthocyanidins, and theaflavins), isoflavones and anthocyanidins. Polyphenol synthesis occur through the general phenylpropanoid pathway (Fig. 1), in which aromatic amino acids (phenylalanine and tyrosine) give rise to thousands of molecules with a multiple phenol backbone by the action of enzyme superfamilies (ligases, oxygenases, oxidoreductases, transferases, etc.)^6,7^. The initial steps, catalyzed by the phenylalanine ammonia lyase (PAL), cinnamate-4-hydroxylase (C4H) and 4-coumaroyl CoA-ligase (4CL), are necessary for the formation of phenylpropanoid monomers which supply the basis for all resulting phenolic compounds^7^ (Fig. 1).

During strawberry fruit development and ripening, important dynamic fluctuations in phenylpropanoid content are reported^8^. Halbwirth et al.^13^ observed two activity peaks during fruit ripening for most of the enzymes involved in flavonoid biosynthesis. The first peak in immature fruit coincides with the accumulation of astringent tannins (i.e. flavan-3-ols and derived proanthocyanidins), while in the later stages of strawberry ripening, a redirection of flavonoid biosynthesis is observed to flavonols and anthocyanins, in order to increase fruit attractiveness towards seed dispersal animals^8,14^. More than 25 different anthocyanins have been detected in fruits of different strawberry cultivars, although in general the pigmentation of the receptacle is due to glycosylated pelargonidins and a small fraction of glycosylated cyanidins^15^. Phenolic acids and their derivatives also show accumulation patterns depending on fruit developmental stages^8^. While hydroxybenzoic acid derivatives are the dominant class during the first stages of fruit growth, they are progressively substituted by caffeic and ferulic acid hexose derivatives and finally coumaric and sinapic acid derivatives, which are almost solely detected in the red ripe fruit^8^.

In strawberry, a number of studies have analyzed polyphenols levels^14,16–18^; however, the knowledge about the genetic mechanisms underlying these processes is more limited^19–21^. Quantitative trait loci (QTL) mapping in bi-parental populations is a method commonly used to dissect the genetic basis of agronomic traits. In strawberry, the majority of studies on both natural variance and metabolite QTL mapping have focused on primary metabolism^22–25^. These studies have identified genomic regions underlying sugar content and titratable acidity, vitamin content and variation in other primary metabolites including organic acids and amino acids in fruits. Targeted QTL analyses have also been performed on volatile organic compounds in strawberry fruit^26,27^. Screens of natural variance have additionally focused on a similar range of compounds^14,28^. Moreover, some of these studies were able to identify candidate genes encoding biosynthetic enzymes affecting these important fruit quality traits.

To date a very limited number of studies have aimed the detection of QTLs affecting the content of bioactive and nutritional compounds in strawberry. Only very recently genomic regions affecting the quantitative variation of fruit flavonoids have been identified in the cultivated strawberry^20,21^. In those studies, the content of 13–21 polyphenol compounds was determined and QTL controlling their variation were detected. In this study, a broader scale analysis of fruit polar secondary metabolite levels was performed in two independent harvests on the ‘232’ × ‘1392’ F_1_ strawberry population that has been previously characterized for agronomic and fruit quality traits^22,24,26^. The objective of this work is to extend the phenotypic characterization of this population to the polyphenol composition and antioxidant capacity on fruits. First, we measured total polyphenol content (TPC) and fruit antioxidant capacity as a rapid estimation of antioxidant content in strawberry fruits. Next, we performed metabolite analysis using Ultra Performance Liquid Chromatography coupled to Tandem Mass Spectrometry (UPLC-Orbitrap-MS/MS) to tentatively identify and semi-quantify secondary metabolites in the population. A QTL mapping approach was then carried out to detect genomic regions and candidate genes involved in the biosynthesis and regulation of phenylpropanoid-derived metabolites in strawberry fruit. Results are discussed both in the context of the regulation of secondary metabolism in strawberry fruits and with respect to the efficiency of identified markers to assist the nutritional fortification of fruits during crop breeding.

## Results

### Variation in total polyphenol content, antioxidant capacity and content of phenylpropanoid metabolites in the ‘232’ × ‘1392’ population

To analyze the antioxidant content of strawberry fruits, total polyphenol content (TPC) and Trolox equivalent antioxidant capacity (TEAC) were analyzed in the whole F_1_ population and the two parental lines during two consecutive years, 2013 and 2014. Parental line ‘1392’ presented higher TPC and TEAC in both years, although the difference was only significant for TEAC in 2014 (Table 1, see sub-section “Variation in Total Polyphenol Content and Antioxidant Capacity” in the Supplementary Note for a detailed description).

**Table 1:** Total polyphenol content (TPC) and antioxidant capacity (TEAC) in fruits of '232’, 1392’ and F_1_ progeny.

Trait	Year	Lines	Mean	SD	Range	‘232’ versus ‘1392’^a^	Transg.^b^	2013 versus 2014 ^c^	H^2^
TPC (mg gallic acid eq/100 g)	2013	232	65.70	7.33	61.22–74.17	ns			
		1392	75.41	1.22	74.54–76.81				
		F_1_ progeny	67.69	8.42	49.40–89.70		Yes		0.57
	2014	232	67.94	3.98	63.44–71.02	ns			
		1392	79.43	6.23	72.26–83.52				
		F_1_ progeny	62.24	9.5	43.18–81.58		Yes	r = 0.22*	0.59
TEAC (μmol trolox/g)	2013	232	34.63	7.79	27.54–42.97	ns			
		1392	45.71	8.56	36.02–52.24				
		F_1_ progeny	35.41	6.1	24.75–50.67		No		0.48
	2014	232	34.54	5.35	30.85–40.68	*			
		1392	45.32	3.91	42.05–49.65				
		F_1_ progeny	29.29	6.16	14.20–45.04		Yes	ns	0.60

The mean values, standard deviations (SD), range, significance level of the difference between parental lines means, and broad sense heritability (H^2^) of the traits are described.

^a^ns and * indicate values that are not significant or significant at *P* < 0.05, respectively.

^b^Trait segregation was declared transgressive (Transg.) when at least one progeny had a value higher or lower than the highest or lowest parental value by at least the SD of the parents.

^c^Pearson correlation between years.

To focus on the variation on individual secondary metabolites that can contribute to TPC and TEAC, we next evaluated which phenylpropanoid-related metabolite were present in ripe fruits from the two parental lines and their F_1_ progeny by Ultra Performance Liquid Chromatography coupled to Tandem Mass Spectrometry (UPLC-Orbitrap-MS/MS) using the same samples previously profiled for TPC and TEAC. We were able to annotate the chemical structure of 78 metabolites, including 53 flavonoids, 14 hydroxycinnamic acid derivatives, six hydroxybenzoic acid derivatives, and five terpenoids. The flavonoid class included 21 condensed tannins or proanthocyanidins (10 propelargonidin and 11 procyanidin oligomers), six flavan-3-ols, 16 flavonols, three flavanones, six anthocyanins and one flavone (Table 2; Supplementary Table S1).

**Table 2:** Metabolites tentatively identified by UPLC-Orbitrap-MS/MS, retention time (RT), molecular ion (m/z), mode (positive/negative), mean value of parental line ‘232’, range in the population and broad sense heritability (H^2^) for 2013 and 2014 harvests.

Metabolite	RT	m/z	Mode (−/+)	‘232’ parental		2013		2014		2013	2014
				2013	2014	Min	Max	Min	Max	H^2^	H^2^
**Procyanidins**											
Procyanidin dimer1	4.66	577.13	–	**1.47**	**2.02**	0.43	1.78	0.62	2.81	0.38	0.30
Procyanidin dimer2	4.89	577.13	–	**1.46**	1.54	0.46	1.72	0.49	3.61	0.43	0.31
Procyanidin dimer3	6.23	577.13	–	1.25	**1.69**	0.52	1.68	0.62	2.78	0.36	0.25
Procyanidin trimer1	3.62	865.20	–	**1.44**	**1.99**	0.45	1.90	0.61	2.87	0.39	0.30
Procyanidin trimer2	5.01	865.20	–	**1.62**	1.46	0.44	2.13	0.44	3.50	0.51	0.29
Procyanidin trimer3	5.10	865.20	–	1.29	**2.03**	0.44	1.84	0.60	2.75	0.40	0.24
Procyanidin trimer4	5.20	865.20	–	1.20	**1.49**	0.48	1.97	0.61	2.81	0.46	0.28
Procyanidin tetramer1	4.46	1153.26	–	**1.56**	**2.58**	0.00	2.84	0.21	5.11	0.56	0.23
Procyanidin tetramer2	4.77	1153.26	–	**1.67**	**2.13**	0.39	2.24	0.42	3.70	0.44	0.28
Procyanidin tetramer3	5.18	1153.26	–	1.09	**1.75**	0.39	1.96	0.55	2.82	0.40	0.26
Procyanidin tetramer4	5.45	1153.26	–	**1.50**	1.22	0.26	2.55	0.24	4.75	0.53	0.25
**Propelargonidins**											
Propelargonidin dimer1	5.24	561.13	–	1.07	1.46	0.38	2.24	0.44	2.64	0.46	0.37
Propelargonidin dimer2	5.57	561.14	–	**0.69**	0.58	0.22	1.84	0.00	3.45	0.55	0.41
Propelargonidin dimer3	6.52	561.14	–	0.82	0.84	0.00	2.08	0.00	6.24	0.34	0.52
Propelargonidin dimer4	6.87	561.14	–	0.97	1.22	0.38	2.31	0.35	2.66	0.53	0.36
Propelargonidin trimer1	3.99	849.20	–	1.39	**1.56**	0.38	1.96	0.39	2.74	0.49	0.46
Propelargonidin trimer2	5.41	849.20	–	1.41	**2.28**	0.00	2.45	0.00	4.33	0.33	0.20
Propelargonidin trimer3	5.46	849.20	–	1.63	1.27	0.00	3.34	0.00	4.32	0.41	0.13
Propelargonidin trimer4	5.63	849.20	–	1.24	**2.23**	0.46	1.73	0.55	2.98	0.44	0.34
Propelargonidin trimer5	6.50	849.20	–	1.45	**2.35**	0.26	2.35	0.23	3.87	0.49	0.36
Propelargonidin trimer6	6.85	849.20	–	1.34	**1.57**	0.38	2.56	0.33	3.16	0.56	0.43
**Flavan-3-ols**											
(Epi)catechin	5.06	289.07	–	1.21	1.59	0.45	1.63	0.57	3.28	0.40	0.31
(Epi)afzelechin1	7.53	435.13	–	**0.24**	**0.23**	0.12	1.13	0.17	1.35	0.64	0.57
(Epi)afzelechin2	8.29	435.13	–	1.18	1.01	0.24	3.20	0.32	3.25	0.74	0.46
(Epi)afzelechin3	8.80	435.13	–	1.23	1.46	0.00	3.26	0.00	4.13	0.37	0.44
Epicatechin glucuronide1	5.17	465.11	–	**0.41**	**0.29**	0.00	1.00	0.05	1.94	0.67	0.48
Epicatechin glucuronide2	5.41	465.10	–	**0.47**	**0.35**	0.04	0.94	0.04	1.89	0.61	0.44
**Flavonols**											
Kaempferol-hex.1	5.17	447.09	–	**0.48**	**0.32**	0.06	1.00	0.08	1.86	0.64	0.46
Kaempferol-hex.2	5.51	447.09	–	**0.13**	**0.12**	0.04	2.83	0.04	3.58	0.87	0.50
Kaempferol-hex.3	7.72	447.09	–	**1.72**	**1.67**	0.29	2.47	0.31	2.81	0.35	0.14
Kaempferol-glucuronide1	7.72	461.07	–	**0.86**	0.99	0.11	1.50	0.21	2.91	0.45	0.12
Quercetin-glucuronide	7.10	477.07	–	1.33	1.46	0.17	1.21	0.18	3.96	0.38	0.13
Isorhamnetin glucuronide	7.94	491.08	–	**2.92**	**1.87**	0.11	4.37	0.15	6.97	0.72	0.42
Quercetin hex	7.14	463.09	–	0.94	0.81	0.16	1.25	0.05	2.84	0.41	0.26
Kaempferol malonylhex	8.14	535.11	+	**5.03**	**3.33**	0.50	8.25	0.47	9.21	0.61	0.11
Kaempferol coumaroyl hex	7.47	595.16	+	**0.49**	0.80	0.15	2.33	0.16	2.76	0.71	0.25
Kaempferol acetylhex	8.65	491.12	+	1.29	1.29	0.08	2.49	0.00	5.20	0.59	0.29
Quercetin-acetylhexose	7.89	507.11	+	**0.59**	**0.36**	0.15	3.12	0.00	4.44	0.65	0.30
Rutin1	5.59	609.15	–	1.04	0.82	0.07	1.04	0.23	2.70	0.58	0.44
Rutin2	6.91	609.14	–	**0.49**	**0.46**	0.07	1.46	0.01	2.79	0.62	0.50
Kaempferol hex. glucuronide	5.55	623.12	–	0.76	1.06	0.07	1.46	0.24	3.85	0.42	0.19
Kaempferol pentose hex. glucuronide2	9.55	593.13	–	**0.40**	**0.54**	0.00	3.25	0.00	4.35	0.65	0.27
Quercetin malonylhex	7.45	551.10	+	**1.95**	**1.93**	0.16	6.81	0.00	5.42	0.59	0.16
**Flavones**											
Diosmetin acetylhex	8.29	503.12	–	1.27	0.90	0.00	4.37	0.00	4.92	0.70	0.40
**Flavanones**											
Eriodictyol hex.1	5.50	449.11	–	1.17	0.96	0.24	1.49	0.23	3.03	0.56	0.46
Eriodictyol hex.2	6.04	449.11	–	**0.89**	0.92	0.20	1.40	0.22	2.81	0.47	0.46
Naringenin chalcone hex.3	6.29	433.11	–	1.03	0.94	0.15	1.89	0.33	2.18	0.40	0.24
**Anthocyanins**											
Cyanidin hexose	5.12	449.11	+	**0.50**	**0.45**	0.05	1.06	0.06	1.86	0.60	0.40
Pelargonidin hex	5.50	433.11	+	0.90	0.87	0.27	1.29	0.46	1.89	0.55	0.43
Pelargonidin malonyl hex	6.37	519.11	+	**2.19**	**1.45**	0.28	7.45	0.04	8.44	0.59	0.36
Pelargonidin rutinose	5.61	579.17	+	**0.42**	**0.55**	0.26	1.28	0.29	1.44	0.58	0.35
Pelargonidin acetyl hexose1	6.38	473.11	–	2.18	1.84	0.07	7.27	0.24	13.74	0.61	0.32
Pelargonidin acetyl hexose2	6.91	473.11	–	1.55	1.56	0.18	2.77	0.00	6.66	0.62	0.39
**Hydroxycinnamic acid derivatives**											
Caffeic acid hex	0.80	341.11	–	0.95	1.00	0.91	1.14	0.80	1.15	0.00	0.30
Coumaric acid hex.1	5.18	325.09	–	**0.25**	**0.28**	0.16	1.03	0.10	1.31	0.57	0.46
Coumaric acid hex.2	5.43	325.09	–	**0.40**	**0.34**	0.17	1.05	0.11	1.68	0.54	0.52
Ferulic acid hex.1	5.56	355.10	–	0.82	0.75	0.48	2.51	0.34	2.80	0.60	0.51
Ferulic acid hex.2	6.98	355.10	–	0.83	0.95	0.19	5.58	0.00	6.72	0.60	0.44
Ferulic acid hex.3	7.39	355.10	–	0.94	0.60	0.34	5.07	0.12	3.19	0.57	0.33
Sinapic acid hex. derivative1	8.53	385.15	–	**2.07**	0.88	0.18	3.97	0.05	4.76	0.60	0.40
Sinapic acid hex. derivative2	9.78	385.15	–	**0.05**	**0.06**	0.00	1.20	0.01	1.23	0.67	0.57
Sinapic acid hex. derivative3	9.95	385.15	–	**0.52**	0.91	0.10	3.05	0.13	2.28	0.61	0.33
Sinapic acid hex. derivative4	11.27	385.15	–	**0.57**	1.04	0.04	2.56	0.07	3.88	0.63	0.48
Coumaric acid1	5.11	163.04	–	**0.69**	0.57	0.29	1.12	0.20	2.76	0.26	0.24
Coumaric acid2	6.43	163.04	–	**0.16**	**0.10**	0.06	1.46	0.04	1.76	0.73	0.60
Cinnamic acid-hexo1	7.00	311.11	+	0.60	0.42	0.12	10.08	0.00	3.96	0.66	0.35
Cinnamic acid-hexo2	7.39	311.11	+	0.76	0.45	0.19	8.79	0.00	4.43	0.74	0.14
**Benzoic acid derivatives**											
Di-hydroxybenzoic acid hex.1	3.63	315.07	–	1.03	0.85	0.60	4.39	0.26	4.52	0.61	0.29
Di-hydroxybenzoic acid hex.2	4.35	315.07	–	1.46	1.41	0.47	11.19	0.32	3.65	0.86	0.35
Di-hydroxy methyl benzoic acid hex.1	3.82	329.09	–	1.12	0.81	0.41	1.82	0.52	1.93	0.21	0.26
Di-hydroxy methyl benzoic acid hex.2	5.97	329.09	–	1.48	1.87	0.20	2.89	0.42	9.55	0.57	0.43
Di-hydroxy methyl benzoic acid hex.3	6.04	329.09	–	0.92	0.84	0.19	1.45	0.19	3.43	0.43	0.41
1-*O*-protocatechuyl-beta-xylose	4.45	285.06	–	1.12	0.72	0.50	8.40	0.37	2.55	0.75	0.71
**Terpenoid derivatives**											
Triterpenoid-hex.1	10.85	695.40	–	1.08	0.98	0.37	4.67	0.07	4.59	0.64	0.24
Triterpenoid-hex.2	11.06	695.40	–	0.89	0.85	0.19	3.27	0.23	6.44	0.53	0.14
Triterpenoid-hex.3	9.33	711.39	–	0.99	0.79	0.40	1.85	0.44	2.19	0.47	0.27
Sesquiterpenoid hex.1	8.47	463.25	–	0.68	1.29	0.32	5.79	0.23	3.83	0.75	0.52
Sesquiterpenoid hex.2	8.99	463.25	–	**0.36**	**0.39**	0.29	2.17	0.29	1.46	0.67	0.31

Bold values indicate significant difference between ‘232’ and ‘1392’ parental lines (*P* < 0.05). Data are relativized to the mean value of the ‘1392’ parental line.

### Hierarchical cluster analysis of phenylpropanoid metabolites in the ‘232’ × ‘1392’ population

**Figure 2:**
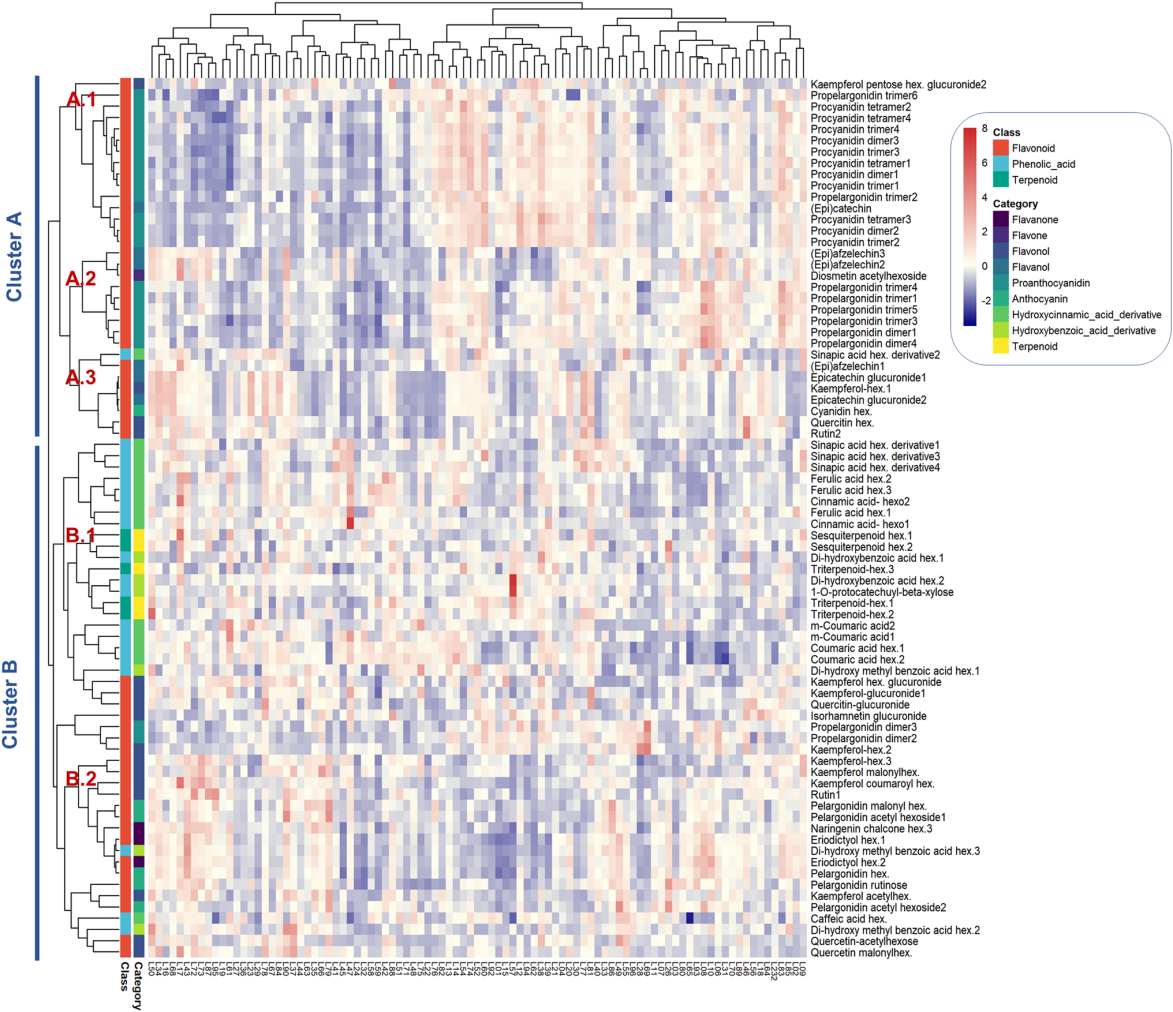
Visualization of the tentative identified secondary metabolites. Hierarchical cluster analysis (HCA) and heatmap visualization of averaged metabolite profiles over two successive years (2013–2014). The lowest response is showed in blue, while the highest response is in red. The exact values for each metabolite peak are provided in Supplementary Table S1.

Figure 2 provides a hierarchical cluster analysis (HCA) from samples harvested in the 2013 and 2014 years. Similar to the observation for primary metabolites^24^, here the range in metabolite contents in the F_1_ progeny was larger than that seen between the two parental lines, ranging from 0 content of metabolite in some F_1_ lines to 13.74-fold change compared to the ‘1392’ parent. As can be observed in Fig. 2, metabolites were grouped into two main clusters (A and B), illustrating biochemical relations among strawberry secondary metabolism (see sub-section “Hierarchical Cluster Analysis of Phenylpropanoid Metabolites in the ‘232’ × ‘1392’ Population” in Supplementary Note for detailed metabolite relations description).

### Correlation analysis of phenylpropanoids

**Figure 3:**
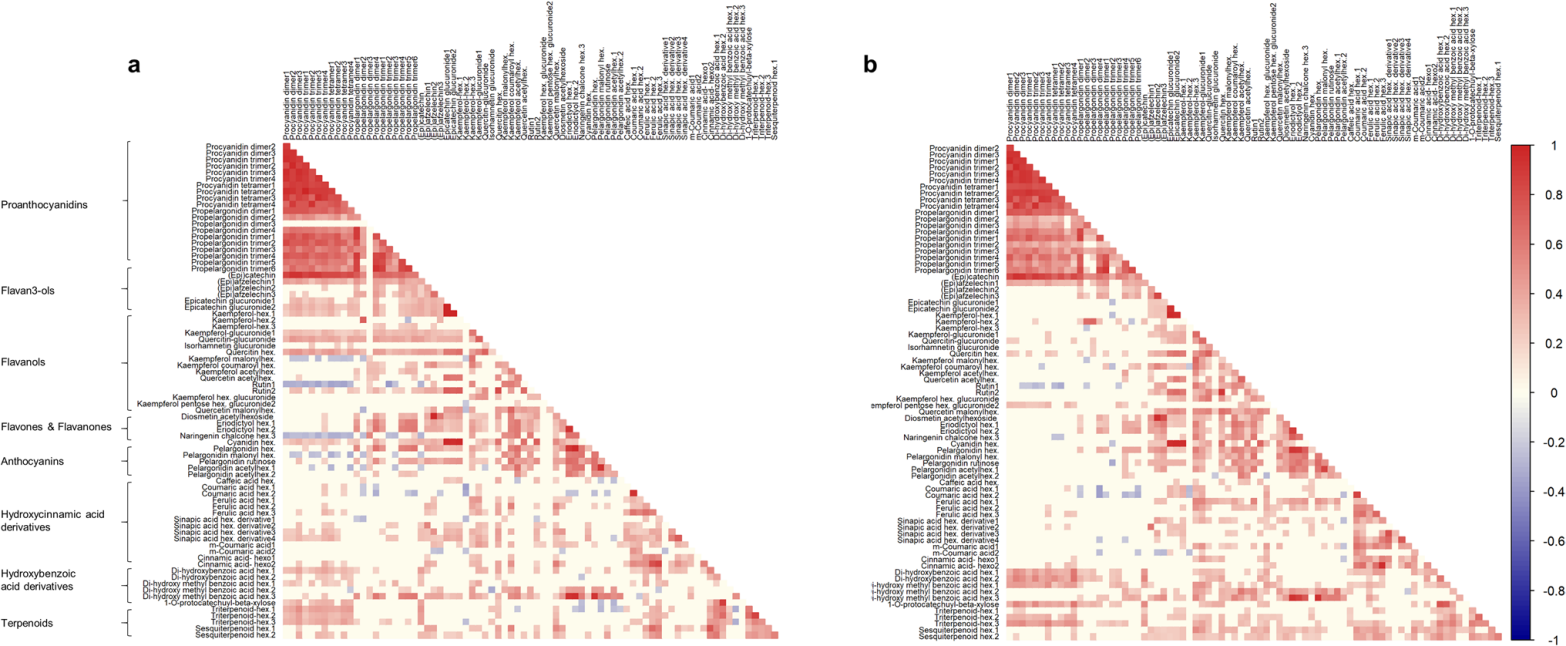
Visualization of metabolite-metabolite correlations. Heat map of metabolite-metabolite correlation between metabolites identified in ‘232’ × ‘1392’ for years 2013 (**a**) and 2014 (**b**). Each square indicates a given r value resulting from Pearson correlation analysis in a false color scale (see color key at the right). Correlation coefficient scores are given in Supplementary Table S2.

Next, we carried out a correlation analysis for all metabolite pairs across the whole spectrum of combinations (Fig. 3; Supplementary Table S2) for the two years (2013 and 2014) separately. A total of 1308 significant correlations (*P* < 0.05) were found for 2013 harvest (Fig. 3a; Supplementary Table S2). A similar result (1241 significant correlations) was obtained for the 2014 harvest (Fig. 3b; Supplementary Table S2), further demonstrating the genetic component on fruit metabolite abundance. See sub-section “Correlation Analysis of Phenylpropanoid” in Supplementary Note for detailed information about positive and negative correlations).

### Heritability of phenylpropanoid metabolites

The majority of secondary metabolites showed moderate to high broad sense heritability in both years, ranging from 0 to 0.87 in 2013, and from 0.11 to 0.72 in 2014 (Table 2). High heritability values (H^2^ > 0.5) for both seasons were shown for (epi)afzelechin 1, kaempferol-hexose 2, rutin 2, coumaric acid-hexose 2, ferulic acid-hexose 1, sinapic acid-hexose 2, coumaric acid 2, 1-*O*-protocatechuyl-beta-xylose and sesquiterpenoid hexose 1 (Table 2). The average broad sense heritability of secondary metabolites was higher in 2013 (0.54) than in 2014 (0.35).

### Detection of QTLs for fruit antioxidant capacity and phenylpropanoid metabolites over two years

QTL analyses for the complex traits TPC and TEAC, and for phenylpropanoid metabolites were performed essentially as described for detection of mQTLs for primary metabolites^24^, using the same fruit samples collected in years 2013 and 2014. Therefore, the detection of mQTLs for polar secondary metabolites extended previous characterization of QTL controlling fruit quality using the ‘232’ × ‘1392’ F_1_ population^22,24,26^. QTLs detected slightly below the threshold by rMQM were included if a significant association between marker and trait was observed by the Kruskal–Wallis test (*P* > 0.005), or if a QTL was detected in the same genomic region for the same metabolite in the other analyzed year or for another related metabolite in any of the analyzed years.

**Figure 4:**
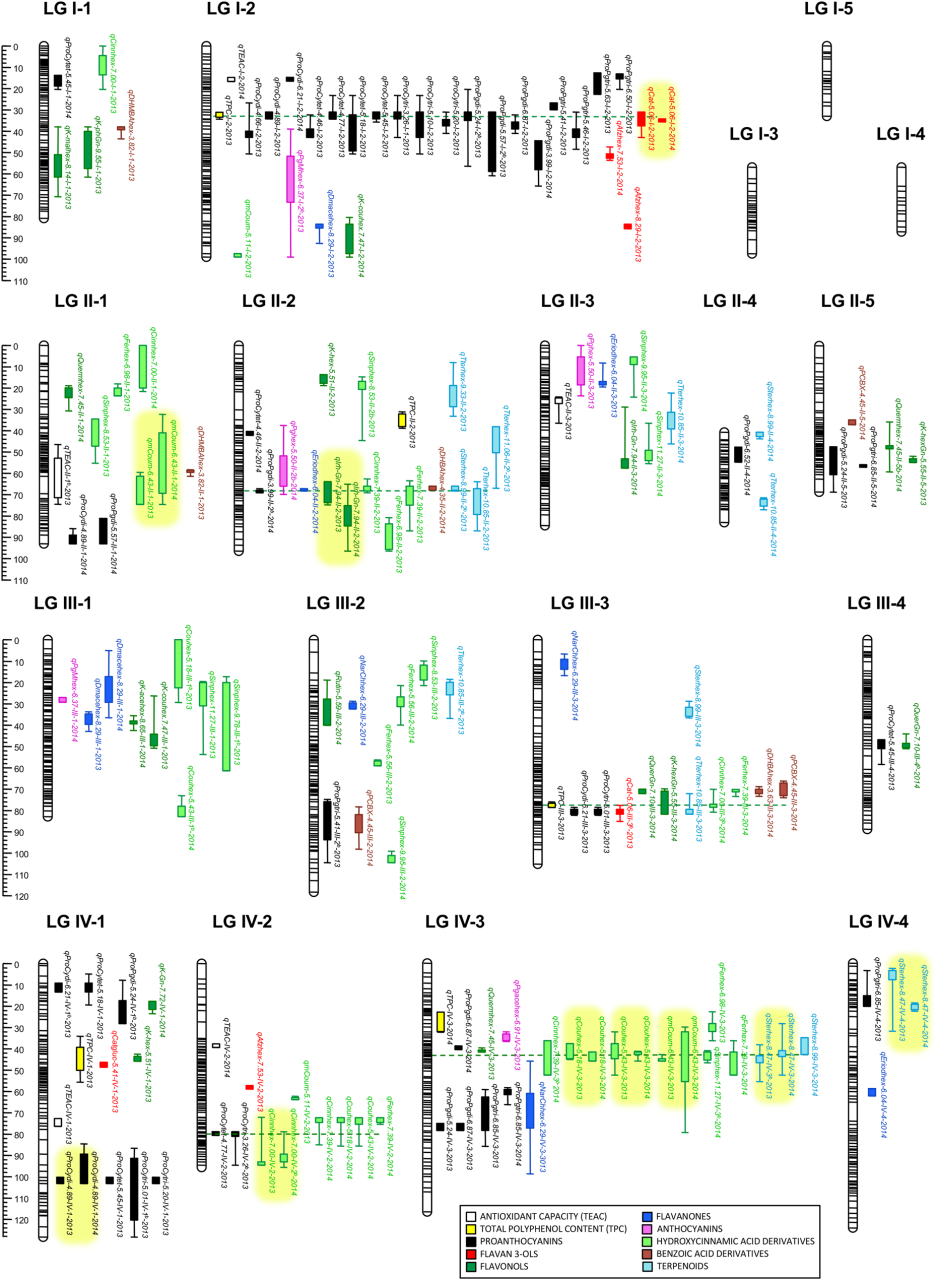

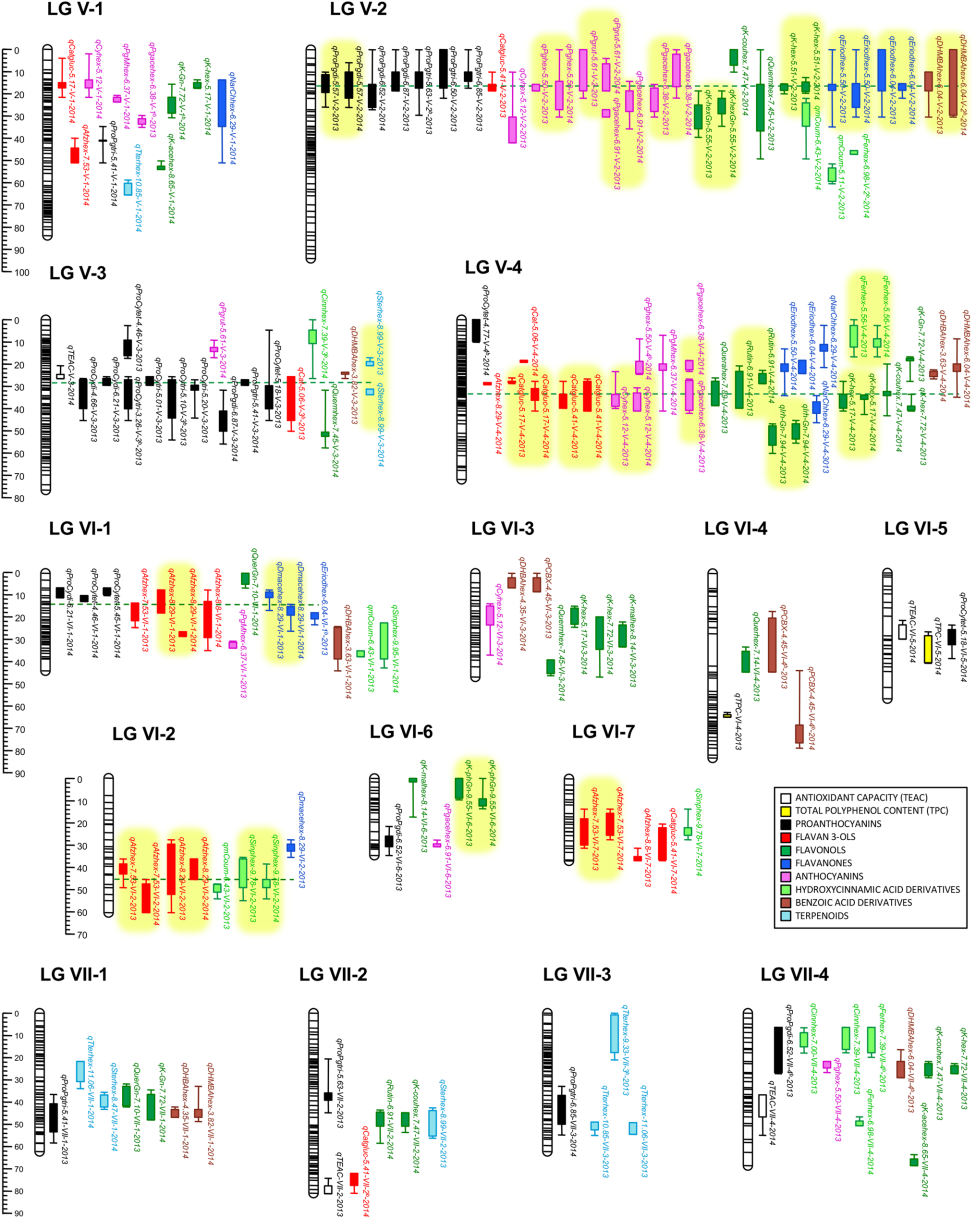
Positions of mQTL controlling phenylpropanoid-derived metabolites and QTL for TPC and TEAC detected in the ‘232’ × ‘1392’ F_1_ population in 2013 and 2014. QTL are indicated by boxes (1-LOD interval) and extended lines (2-LOD intervals). Names of mQTLs are described in Supplementary Table S3. Major QTL (mean R^2^≧ 20%) detected in both years are highlighted in yellow. Putative pleiotropic QTL are joined by green discontinued lines.

In total, we detected 7 QTLs controlling TPC (5 in year 2013 and 2 in 2014). None of them was detected during both years (Fig. 4; Supplementary Table S3). Similarly, 4 and 5 QTLs for TEAC were detected in 2013 and 2014, respectively, and all of them were observed only in one of the two years. Two QTLs controlling TPC, *qTPC-IV-1-2013* and *qTPC-IV-3-2014*, were detected at approximately the same genomic regions in two homoeologous linkage groups (LGs) belonging to homoeology group IV (Fig. 4). These QTLs could therefore be considered as putative homoeo-QTLs. One QTL controlling TPC and one controlling TEAC in 2014 overlapped in LG VI-5, which might indicate the presence of a locus controlling both traits. However, the mean effects associated to each allele differed between the two QTLs (Supplementary Table S3), suggesting the presence of two linked loci with positive alleles for TPC and TEAC coming from the male and female parent, respectively.

A total of 309 mQTLs for 77 out of the 78 metabolites were detected over the two years (Fig. 4; Supplementary Table S3). Among them, 41 QTLs were detected in the same chromosomal regions (with overlapping confidence intervals) over the two years and were considered stable, while 227 mQTLs were detected only on 1 year. Therefore, only 15.3% of the total 268 mQTLs were stable over the two years. The majority of these stable mQTLs (36 out of the 41) controlled a large proportion (an average R^2^ > 20% over the two years) of phenotypic variance (highlighted in yellow in Fig. 4; Supplementary Table S3).

mQTLs controlling phenylpropanoid-derived metabolites were detected in all linkage groups, with the exception of the three short LGs I-3, I-4 and I-5. The average number of QTLs per metabolite was 4 and ranged from one (i.e. for quercetin hexose, rutin isomer 1 and di-hydroxy methyl benzoic acid hexose 2) to eight for (epi)afzelechin isomer 1. The average phenotypic variance (R^2^) explained by each mQTL was 22.9% and ranged from 3.5% (for *qK-acehex-8.65-III-1* in 2014) to 66.6% for *qK-hex-5.51-V-2* in 2014. Overall, a mean of 46.4 QTLs were detected per homoeology group (HG). The HG with less QTLs controlling antioxidant compounds was HG VII with 28 (8.62%) while 92 QTLs (28.3%) were detected in HG V. The average number of QTL per LG was about 10, although LGs I-2, IV-3, V-2 and V-4 doubled the number of QTLs (28, 22, 32 and 31 QTL, respectively). Interestingly, clusters of QTLs for related metabolites or for different isomers of the same metabolite were detected in those four LGs and others, suggesting the presence of loci controlling the levels of antioxidant compounds in a coordinated way (Fig. 4). See sub-section “Clusters of QTL for Phenylpropanoid Metabolites” in Supplementary Note for detailed description of QTL clusters detected for proanthocyanidins, hydroxycinnamic acid derivatives, terpenoids and (epi)afzelechin. The largest hotspots for flavonoid compounds were located on LG V-2 and V-4, and included many stable mQTLs with major effects on metabolite variance (Fig. 4; Supplementary Table S3). On LG V-2, stable mQTLs were detected for the anthocyanins pelargonidin-hexose (the main pigment in strawberry), pelargonidin acetyl hexose and pelargonidin rutinose, for derivatives of the flavonol kaempferol and for derivatives of the flavanone eriodictyol. Similarly, another cluster of mQTLs including similar range of flavonoids was observed in LG V-4, with stable QTLs for the anthocyanins pelargonidin acetyl hexose 1 (also detected on LG V-2) and cyanidin hexose, the two isomers of the flavanol epicatechin glucuronide, for the flavonols isorhamnetin glucuronide, kaempferol hexose 1 and rutin 2, and the flavanone naringenin chalcone hexose.

### Candidate genes at mQTL hotspots on LG V-2 and LG V-4

Hotspot genomic regions with major mQTL on LG V-2 and V-4 were selected for candidate gene identification based on colocalization to the *F. vesca* and/or *F.* × *ananassa* ‘Camarosa’ genomes^29–31^. Several mQTLs for flavanones, flavonols, anthocyanins, flavan-3-ols and propelargonidins collocated on those two genomic regions. No reported gene encoding for any enzyme of the flavonoid pathway were located on the *F. vesca* reference genomic regions between markers flanking the QTL confidence intervals. However, a number of predicted genes with similarity to structural genes or transcription factors were detected. Interestingly, the transcriptional repressor *FaMYB1* (*FvH4_5g17120*), is located within the *F. vesca* orthologous region to the QTL hotspot interval in LG V-4. *FaMYB1* is a repressor of anthocyanin and flavonol biosynthesis in strawberry^32,33^. A search on the octoploid strawberry genome^31^ identified 5 copies of *FaMYB1* in the ‘Camarosa’ reference genome, with one copy on chromosomes Fvb5-1 (*FxaC_17g22290*), Fvb5-2 (*FxaC_20g18010*) and Fvb5-4 (*FxaC_19g15290*) and two copies on chromosome Fvb5-3 (*FxaC_18g28140* and *FxaC_18g28180*). *FaMYB1* homoeologous genes in Fvb5-1 and Fvb5-3 (the corresponding ‘Camarosa’ chromosomes to LG V-2 and V-4 in the ‘232’ × ’1392’ map, respectively) were located at 10.50 and 18.37 Mb, while confidence intervals of the mQTL hotspots detected on LG V-2 and LG V-4, spanned 2.67–7.70 and 17.55–20.10 Mb regions, respectively. Therefore, only the two copies of *FaMYB1* on chromosome Fvb5-3 collocate with mQTL hotspot on LG V-4, while the copy on Fvb5-1 lies outside of the QTL interval on LG V-2.

The gene *FaF3′H* (*FvH4_5g14010*), encoding the flavonoid 3′-hydroxylase enzyme is located just outside the genomic region corresponding to the confidence intervals in LG V-2 and V-4 both in *F. vesca* and *F.* × *ananassa* genomes. The F3′H enzyme catalyzes the hydroxylation of flavonoids up-stream of DFR^34^ (Fig. 1). There are three copies of *FaF3′H* in the octoploid genome, on chromosomes Fvb5-1, Fvb5-2 and Fvb5-3, with *FaF3′H* genes *FxaC_17g18710* and *FxaC_18g31790* located close to the QTL hotspots on LG V-2 and LG V-4 at positions 8.87 and 20.27 Mb, respectively.

### Expression of *FaMYB1* and *FaF3′H* genes in F_1_ lines contrasting in flavonoid content

To investigate the involvement of *FaMYB1* and *FaF3′H* in the variation of flavonoids in hotspots on LG V-2 and LG V4, we analyzed the expression of both genes in contrasting F_1_ lines. For hotspot on LG V-2, we focused the analysis to the metabolites corresponding to the 10 stable mQTLs (highlighted in yellow in Fig. 4). Among them, two groups of metabolites could be distinguished based in significant correlations: (1) propelargonidin dimer 2 and kaempferol hexose 2 and (2) the rest of metabolites (pelargonidin derivatives, eriodictol hexose 1 and 2 and the benzoic acid derivative) with the exception of kaempferol hexose glucuronide. Strikingly, kaempferol hexose glucuronide was not significantly correlated to any of the two groups. Each of these two groups contained secondary metabolites with high positive correlations among them in the two years, as example propelargonidin dimer 2 and kaempferol hexose 2 had a correlation coefficient of ~ 0.7 in both years (Fig. 3; Supplementary Table S2). In addition, the mQTL for metabolites in each group displayed the same direction of allelic effects: a positive allele (increasing the concentration) was inherited from the female and male parent for the (1) and (2) groups, respectively. Together, these data suggest that the variation of each group of metabolites might be controlled by a different gene. Therefore, two groups of contrasting lines, (V-2-1) and (V-2-2), each composed of two pools of six lines, were selected based on the concentration of metabolites belonging to these two groups for differential expression analysis (Supplementary Table S4).

Among the metabolites affected by the major mQTLs in LG V-4 hotspot, epicatechin glucuronide isomer 1 and 2, kaempferol hexose 1, cyanidin hexose and rutin isomer 2 displayed high positive correlations (Fig. 3; Supplementary Table S2). Except for rutin 2, correlations between these metabolites were higher than 0.9 in both years. The correlation coefficients for rutin 2 with the rest of metabolites ranged from 0.6 to 0.7. In addition, positive alleles for the major mQTLs controlling the variation of these five metabolites were in all cases inherited from both parental lines, suggesting the presence of a common locus in heterozygosis in both parental lines controlling their variation. Therefore, pools of contrasting lines with high and low content of these five flavonoids (V-4) were selected for expression studies. Selected contrasting lines with their relative metabolite content, as well as the genotypes of markers on the mQTL intervals are shown on Supplementary Table S5.

**Figure 5:**
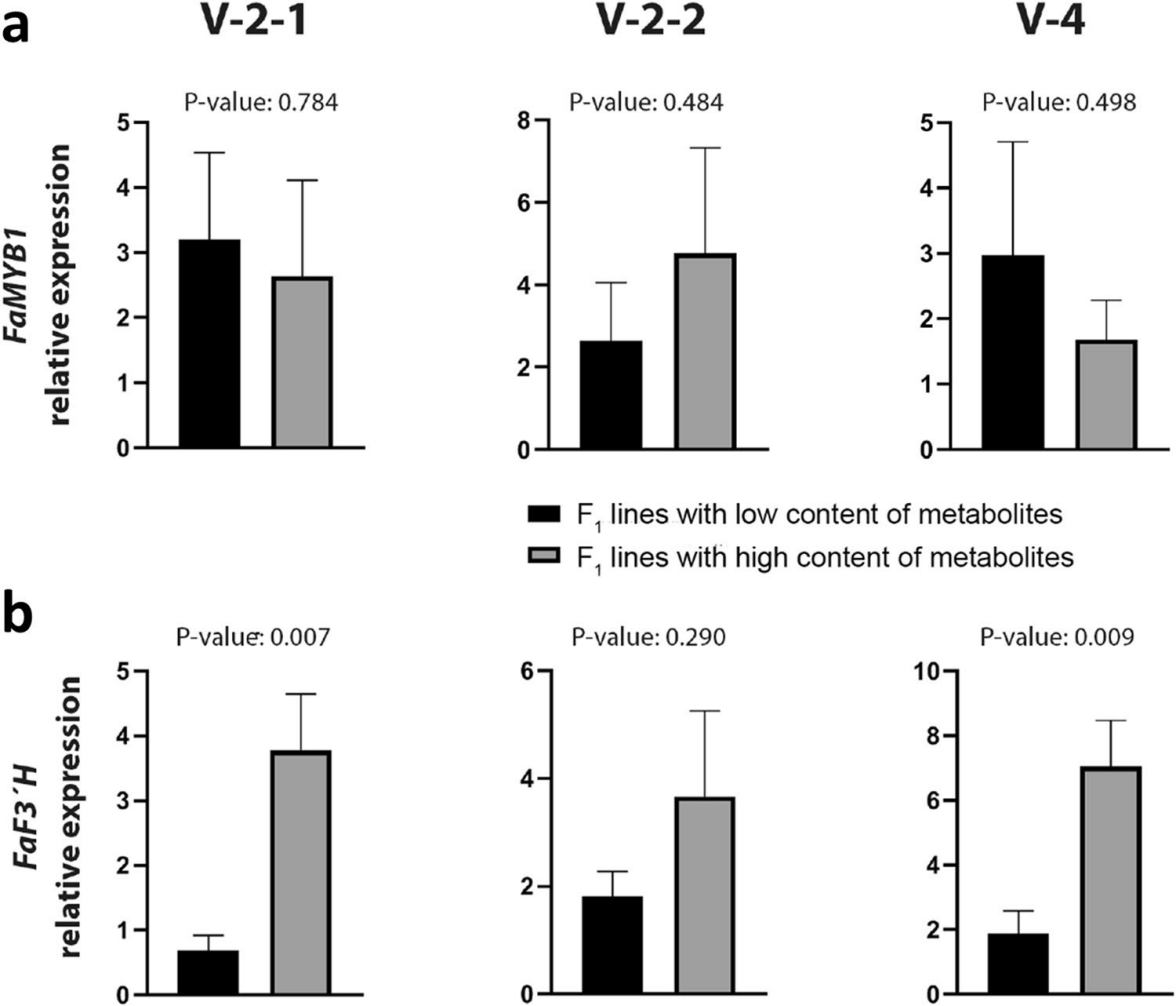
Expression analysis by quantitative real-time PCR (qRT-PCR) of *FaMYB1* (**a**) and *FaF3′H* (**b**) genes in selected pools of ripe fruit contrasting in metabolite content (low and high content in black and grey respectively). Metabolites in V-2-1 are propelargonidin dimer 2 and kaempferol hexose 2. Metabolites in V-2-2 are pelargonidin acetyl hexose 1 and 2, pelargonidin hexose, pelargonidin rutinose, eriodictol hexose 1 and 2 and di-hydroxy methyl benzoic acid hexose 3. Metabolites in V-4 are epicatechin glucuronide isomer 1 and 2, kaempferol hexose 1, cyanidin hexose and rutin isomer 2. Error bars indicate + SD of six F_1_ lines with three biological replicates (n = 18). Significant differences in expression between high and low pools were analyzed using a *t*-Student test.

Quantitative real time PCR (qRT-PCR) analyses in selected pools were carried out to compare *FaMYB1* transcript levels in high and low accumulating lines (Fig. 5a). No significant differences were observed between contrasting lines for any metabolite. In contrast, significant differences in *FaF3′H* expression were observed between pools contrasting in the content of metabolites in V-2-1 and V-4 (Fig. 5b). Thus, lines with high levels of propelargonidin isomer 2, kaempferol hexose 2 (V-2-2), epicatechin glucuronide isomer 1 and 2, kaempferol hexose 1, cyanidin hexose and rutin isomer 2 (V-4) showed significantly higher expression of *FaF3′H*, suggesting that this candidate gene might be controlling natural variation of these related flavonoids in strawberry.

## Discussion

Total polyphenol content and antioxidant capacity in fruits and vegetables are common parameters for estimation of its health benefits. However, both traits represent aggregated or composite traits gathering the variation of many different antioxidant metabolites, such as polyphenolic compounds and vitamins such as ascorbic acid^35^. In this work, both composite traits, TPC and TEAC, and the majority of phenylpropanoids-related metabolites appear to be under strong environmental control. Thus, all QTL controlling TPC and TEAC and 84.3% of mQTL controlling individual metabolites were only detected during one of the two assessed years. Similar results were recently reported by Labadie et al.^20^ in a different F_1_ population of octoploid strawberry, in which the authors detected low stability of QTLs for flavonoids in ripe fruits. Due to their function protecting against oxidative stress from several environmental factors (e.g. pathogens, water stress, temperature, and light), the environment plays a major role in inducing antioxidant metabolites^7^. Consequently, it is expected that the genetic effect of composite traits such as TPC and TEAC may not be as strong as the environmental effect along different harvests. The variation observed can also be due to plant-to-plant variability within the three biological replicates, as observed in particular for the TPC and TEAC values, where standard deviation (SD) was in several genotypes, i.e. parental lines, as high as the SD observed among the F_1_ progeny (Table 1). Similarly, other studies in strawberry and tomato have reported strong environmental effects over polyphenols and antioxidant capacity^19,20,36–38^. Nevertheless, 15.3% of mQTLs controlling secondary metabolite content were detected in the two assessed years. Furthermore, broad sense heritability for TPC, TEAC and the majority of secondary metabolites in strawberry fruit was moderate, suggesting an important genetic component. This agrees with results in Arabidopsis and tomato where secondary metabolites showed higher heritability than primary metabolites^39,40^. Weak correlation between different flavonoids and antioxidant capacity has been recently reported in an independent study in strawberry fruit^20^. Taken together, all these data indicate that marker-assisted selection (MAS) for increasing TPC and TEAC in strawberry fruit may not be feasible, and instead, breeding strategies for antioxidant content improvement should be directed to specific metabolites with mQTLs controlling large amount of variance and stable in different years.

A great variation on phenylpropanoid derived metabolite content was observed across the population, with the majority of them showing more than tenfold change. Although the variation for primary metabolites across this population was also high^24^, a larger variation was in general observed for secondary metabolites. Furthermore, a large proportion of secondary metabolites were not detected (concentrations below the detection limit) in a number of F_1_ lines while showed high levels in other lines. Transgressive segregation was generally observed towards lower values. A similar range of variation and both environment and genetic effects have also been observed in the wild diploid *Fragaria vesca* introgressed with *F. bucharica* and in a population of the octoploid strawberry^19,20^.

HCA and metabolite-metabolite correlations highlighted co-regulation of metabolites based on their biochemical relationships. As expected, strong positive correlations were observed within common metabolic classes, such as proanthocyanidins and flavan-3-ols precursors, or among hydroxycinnamic acid derivatives. Correlation of different or related metabolites in combination with detection of epistatic mQTLs (in QTL hotspots) can help in deciphering which pathway(s) or enzyme(s) is likely involved in affecting their natural variation. Surprisingly, few negative correlations were observed between different classes. Interestingly, some negative correlations were observed between different flavonoids and hydroxycinnamic acid derivatives. Indeed, competition between flavonoid and lignin biosynthesis for a common substrate (coumaroyl-CoA) has been previously reported in strawberry^28,41,42^.

QTLs controlling secondary metabolites were well spread across the strawberry genome. However, in several instances, hotspots of mQTLs were detected, which might suggest the presence of a locus controlling several related metabolites. Interestingly, the majority (28 out of 36) of mQTLs that were stable over the two years and controlled a large proportion (an average R^2^ > 20% over the two years) of phenotypic variance were clustered in hotspots on LG IV-3, LG V-2 and V-4, and LG VI-1 and VI-2 (highlighted in yellow in Fig. 4; Supplementary Table S3). Loci on those regions and markers associated represent useful targets for marker assisted selection of new varieties with increased levels of antioxidant compounds. Detailed information about putative underlying candidate genes on LG IV-2, LG IV-3 and LG I-2 is discussed in Supplementary Discussion.

The largest clusters of mQTLs were detected in LG V-2 and V-4. Detailed analysis of correlations between metabolites and between the QTL allelic effects suggested the presence of at least two linked loci in each hotspot controlling subsets of phenylpropanoid metabolites. Two candidate genes encoding a well-known transcription factor, *FaMYB1*, and a flavonoid biosynthetic gene, *flavonoid-3′-hydroxylase* (*FaF3′H)*, were identified within or close by the QTL hotspots. *FaMYB1* is a repressor of anthocyanin and flavonol biosynthesis in strawberry^32,33^. Reduction of *FaMYB1* expression by RNAi resulted in increased expression of *ANR* and *LAR* transcripts and accumulation of flavan-3-ols, which might affect proanthocyanidin accumulation^32^. However, no significant differences in the expression level of *FaMYB1* was found between pools of lines contrasting in the content of these flavonoids, dismissing this gene from being the underlying candidate, at least based on gene expression. We cannot yet formally exclude this TF as candidate gene, and ORF sequence analyses and more detailed gene expression studies may allow to address this hypothesis more comprehensively.

The gene *flavonoid-3′-hydroxylase* (*F3′H*) is located very close to mQTL hotspots in LG V-2 and V-4. Expression analysis of F_1_ lines contrasting in the levels of propelargonidin dimer 2, cyanidin hexose, epicatechin glucuronide 1 and 2, the flavonols kaempferol hexose 1 and 2, and rutin 2 showed that high expression of *FaF3′H* is associated with high content of these flavonoids. The activity of F3*′*H on early compounds determines the 3*′*-hydroxylation pattern of the B-ring in downstream flavonoid compounds^43^. Therefore, increased levels of dihydroxylated flavonoids (cyanidin, epicatechin and rutin), which variation is largely controlled by the mQTL in LG V-4, is expected in lines with higher *FaF3′H* expression. However, why monohydroxylated flavonoids such as propelargonidins or kaempferol hexose accumulate to higher levels in lines with higher *FaF3′H* expression needs further investigation. One possibility could be that FaF3′H had higher substrate specificity for dihydrokaempferol than for kaempferol. Different substrate specificities have been reported for other enzymes in the flavonoid biosynthetic pathway such as DFR^44^ or 4CL enzymes^45^.

Cultivated strawberry fruits display a prevalence of monohydroxylated flavonoids (i.e. pelargonidins) in comparison to other berry fruits and to the wild *F. vesca*, which accumulate a higher amount of dihydroxylated flavonoids such as cyanidins and quercetin^14,43^. Direct proof concerning the mechanisms underlying the partition across these metabolites is still unknown. However, our results suggest that variation in the candidate *FaF3′H* gene might be the underlying molecular mechanism affecting their natural variation in octoploid strawberry. Interestingly, an eQTL controlling the expression of *FaF3′H* has been detected in a wider collection of cultivars^46^, further indicating an extensive natural variation in the transcript levels of this gene in ripe fruit.

No association between transcript levels of *FaMYB1* or *FaF3′H* and the variation in several pelargonidin derivatives, eriodictol hexose 1 and 2 and the benzoic acid derivative was detected in our study (V2-2 in Fig. 5). The mQTLs in the hotspot of LG V-2 associated with the variation in these flavonoids controlled a large amount of the observed phenotypic variance (Supplementary Table S3 and S4). Among these metabolites, pelargonidin derivatives are the main anthocyanins that contribute to strawberry fruit color. In agreement, QTLs for color related traits were detected in the same genomic region of LG V-2 in a previous study using the same population^22^. Although no candidate gene has been identified yet in this region, markers linked to these QTLs, such as ChFaM044, M00247-47:C>G and M30873-45:T>G (Supplementary Table S4), here identified represent useful tools for accelerating breeding of fruit color in octoploid strawberry.

## Methods

### Plant material

The mapping population used consists of a full-sibling family of 95 F_1_ individuals derived from an intraspecific cross between the breeding lines ‘232’ and ‘1392’, and has been previously characterized for agronomic and fruit quality traits^22^. Six plants of each F_1_ and parental lines were vegetatively propagated and grown under commercial conditions in Moguer (Huelva, Spain) during two consecutive years (2013–2014). The mapping population was grown under macro tunnels of polyethylene with an inter-row distance of 30 cm and a distance between plants of 25 cm. Variation in agronomical, fruit quality traits and volatiles have previously been described for years 2007, 2008 and 2009^22,26^, and variation in primary metabolite traits in ripe fruits has also been characterized for the same years used in this study^24^. About 25 fully mature fruits from all F_1_ individuals and parental lines were harvested the same morning in one day at the pick of the harvest of the two consecutive seasons. Per line, all selected fruits were phenotypically similar for shape, size, firmness and colour. Fruit from each line was pooled into three biological replicates, immediately frozen, ground in liquid nitrogen, and stored at − 80 °C until analysis.

### Extraction and quantification of total polyphenol content and antioxidant capacity

For extraction of polyphenols, 0.5 g of frozen powder from fully ripe strawberry fruit was homogenized in 1 ml of acetone:acetic acid (99:1 v/v) solution using a vortex during 2 min, and then centrifuged at 8000 rpm for 15 min at 4 °C. The supernatants were transferred to vials and stored at − 80 °C until used to determine both total polyphenol content and antioxidant capacity.

Total polyphenol content (TPC) was determined following the Folin-Ciocalteu method^47^. 10 µl of extract was diluted with 175 µl of Milli-Q water and then 12 µl of Folin-Ciocalteu reagent. After 3 min, 30 μl of 20% sodium carbonate solution was added. Samples were incubated for 1 h at room temperature in the dark and then the absorbance at 760 nm was measured in a UV/Vis microplate spectrophotometer (Thermo Scientific Multiskan GO). Gallic acid was used as standard for the calibration curve, and the results were expressed in milligrams of gallic acid equivalents (GAE) per 100 g fresh weight (mg GAE/100 g FW).

Antioxidant capacity of fruit samples was measured by the ability of antioxidant molecules to quench the ABTS^·+^ radical cation [2,2′-azinobis(3-eth-ylbenzothiazoline-6-sulfonate); Sigma-Aldrich] in comparison with the antioxidant activity of standard amounts of Trolox. The Trolox equivalent antioxidant capacity (TEAC) assays were performed as described^48,49^ using 2 μl of extract and 250 μL of radical reagent. The absorbance was measured after 5 min at 25 °C using the above-described spectrophotometer. Results were expressed in μmoles of Trolox equivalents per gram of fresh weight (μmol TE/100 g FW).

### Extraction and analysis of secondary metabolites by UPLC-Orbitrap-MS/MS measurements

Secondary metabolites were extracted using 250 mg frozen material. The extraction procedure was performed as follows: 1.0 ml of cold mixture of methyl-tert-butyl-ether:methanol (3:1) was added and the mixture was sonicated at room temperature for 10 min. Later, 0.65 ml of mixture of water:methanol (3:1) to each vial. The vials were then centrifugated at 4 °C and 10,000*g* for 5 min. A fixed volume (0.6 ml) of polar phase was transferred to a fresh vial before concentrating the extract to dryness in Speed-vac (Centrivac, Heraeus Instrument, Hanau, Germany). Polar secondary metabolites were determined by UPLC-Orbitrap-MS/MS as described in Vallarino et al.^14^. Pre-processing of raw chromatograms was performed using Expressionist Refiner MS 10.0 (GeneData; https://www.genedata.com) with an established workflow. Metabolites were putatively annotated by searching the m/z value against the KEGG compound database^50^. The MS/MS fragmentation of the metabolites was compared with putative molecules found in databases and verified with literature on similar compounds reported in strawberry. Integration of the peak area of the corresponding molecular ion was used to quantify the metabolites in the different lines of the population. Data were expressed as the relative content of each metabolite compared to the ‘1392’ parental.

### Data analysis

Normality of trait distributions was evaluated by the Kolmogorov–Smirnov test. For most metabolites deviating from normality (*P* < 0.05), a number of transformations (Ln, square root, inverse of square root, square, cube, reciprocal, and arcsine) were tested and the transformation that gave the least-skewed result was used to perform QTL analysis. Correlation analysis based on Pearson correlation, student’s t-test, ANOVA and Tukey HSD test were performed on GraphPad Prism 8 or using R software. Broad sense heritability (H^2^ = V_G_/V_P_; V_G_ is the total genetic variance and V_P_ is the total phenotypic variance) was calculated from the variance components obtained by ANOVA as previously described^51^.

### QTL analysis

QTL analysis was conducted in the integrated map previously developed for the ‘232’ × ‘1392’ mapping population using MapQTL 5^52,53^. The population was coded under the population type ‘cross pollinated’ (CP) and QTL analyses performed as reported for primary metabolites^24^. Essentially, the raw relative data from each year were first analyzed by the nonparametric Kruskal–Wallis rank-sum test. A stringent significance level of *P* ≤ 0.005 was used as a threshold to identify markers linked to QTL. Second, raw or transformed data sets for non-normally distributed traits were used for QTL detection using interval mapping (IM) with a step size of 1 cM and a maximum of five neighboring markers. The significant LOD threshold of QTL was determined using a 1000-permutation test for each trait. QTL with LOD scores at the 95% genome-wide threshold were declared significant. Restricted multiple QTL mapping (rMQM) was then performed using the nearest significant markers as co-factors. Significant mQTL location and 1- and 2-LOD confidence intervals were drawn using MapChart 2.2.

### In silico candidate gene search

Physical map positions in *F. vesca* genome of DArT-derived SNPs and microsatellites used in this study were previously obtained by aligning the DArT sequences and SSR primer sequences in the ‘232’ × ‘1392’ map to the most updated *F. vesca* v4.0.a1 genome assembly^54^ using Bowtie 2.2.9 as previously reported^24^. Similarly, physical positions of marker sequences on the recently reported *F.* × *ananassa* genome^31^ were obtained using Bowtie 2.2.9. Candidate genes on the chromosomal regions spanning the positions of markers flanking the 2-LOD confidence interval were searched based on annotated biochemical functions.

### RNA extraction and qRT-PCR

The three different groups of contrasting lines each consisted in six lines with high and six lines with low content of selected metabolites. For each line, three biological replicates consisting of about eight ripe fruits each were analyzed. Total RNA was extracted from strawberry fruits as previously reported^24^. Before reverse transcription, RNA was treated with DNase I (Fermentas) to eliminate contaminating genomic DNA. First-strand cDNA synthesis was performed using 750 ng of RNA in a final volume of 20 μl using the iScript cDNA synthesis kit (Bio-Rad), according to the supplier’s protocol. Relative quantification of transcripts was analyzed by qRT-PCR using the SsoAdvance Universal SYBR Green Supermix (Bio-Rad) and the comparative cycle threshold method. Expression data were normalized to the reference genes *FaCHP1* (*FvH4_7g15070*) and *FaGAPDH2* (*FvH4_4g24420*). *FaMYB1* (*FvH4_5g05100*) transcripts were quantified using primers Forward: GGCGTGGTCGATCCAAGA and Reverse: GCAACCTTCGCCGTGTTTT. Primers used for *FaF3′H* (Forward: CCGTAGCGTCTCAGTTCTTG; Reverse: ACGAGGTCCTGGTAGTTGTA) are described in^55^. Primers used for *FaCHP1* (Forward: TGCATATATCAAGCAACTTTACACTGA; Reverse: ATAGCTGAGATGGATCTTCCTGTGA) are described in^56^. Primers used for *FaGAPDH2* (Forward: TCCATCACTGCCACCCAGAAGACTG; Reverse: AGCAGGCAGAACCTTTCCGACAG^57^.

## Supplementary information

Supplementary Information.

Supplementary Table 1.

Supplementary Table 2.

Supplementary Table 3.

Supplementary Table 4.

Supplementary Table 5.

Supplementary Figure.
