# Supplementary material for: Genetic analysis of phenylpropanoids and antioxidant capacity in strawberry fruit reveals mQTL hotspots and candidate genes
Source: Sci Rep. 2020 Nov 19;10:20197. doi: 10.1038/s41598-020-76946-x (PMC7677386; doi:10.1038/s41598-020-76946-x)
Supplement: Supplementary file 1 — Supplementary Information. [file 41598_2020_76946_MOESM1_ESM.docx]

Supplementary Information

Genetic analysis of phenylpropanoids and antioxidant capacity in strawberry fruit reveals mQTL hotspots and candidate genes

**Delphine M. Pott^1,2^, José G. Vallarino^1,2^, Eduardo Cruz-Rus^2,3^, Lothar Willmitzer^4^, José F. Sánchez-Sevilla^2,3^, Iraida Amaya^2,3^*, Sonia Osorio^1,2^***

^1^Departmento de Biología Molecular y Bioquímica, Instituto de Hortofruticultura Subtropical y Mediterránea “La Mayora”, Universidad de Málaga-Consejo Superior de Investigaciones Científicas, Campus de Teatinos 29071, Málaga, Spain, ^2^Unidad Asociada de I + D + i IFAPA-CSIC Biotecnología y Mejora en Fresa, Málaga, Spain, ^3^Laboratorio de Genómica y Biotecnología, IFAPA Centro de Málaga, 29140 Málaga, Spain, ^4^Max-Planck-Institut für Molekulare Pflanzenphysiologie, Potsdam-Golm, Germany

**Supplementary Note**

**Variation in Total Polyphenol Content and Antioxidant Capacity**

Continuous variation on TPC and TEAC was observed in the F_1_ progeny and normal distributions were noticed in both years (Supplementary Fig. S1). Progeny means for both traits were in general more similar to ‘232’, the parental line with lower content in polyphenols and less antioxidant capacity in fruits (Table 1; Supplementary Fig. S1). Transgressive segregation in F_1_ lines were in general observed for lower TPC and TEAC values than parental lines. The broad sense heritability was moderate for both traits (Table 1), which suggests that genetic components accounted for a significant part of the phenotypic variance for these two composite traits on this population. In addition, TPC and TEAC of fruits appear to be highly influenced by the environment as low or no significant correlation was found between the years (Table 1; Supplementary Fig. S1). However, a significant correlation between TPC in 2013 and TEAC in 2014 was observed, and TPC in 2014 was positively correlated with TEAC in both years (Supplementary Fig. S1). As an additional proof of both genetic and environmental effects over TPC and TEAC, joint analysis of variance (ANOVA) using the data from both years showed that the variations among genotypes, environments, and genotype × environment interactions were all significant (Supplementary Fig. S1).

## Hierarchical Cluster Analysis of Phenylpropanoid Metabolites in the ‘232’ × ‘1392’ Population

In Figure 2, cluster A grouped 32 metabolites, all belonging to the flavonoid class with the exception of one hydroxycinnamic acid (sinapic acid hexose derivative 2). Three subclusters (A.1-3) defined cluster A, the first one (A.1) grouping all procyanidin oligomers together with (epi)catechin and the second one (A.2) most propelargonidin oligomers with two isomers of (epi)afzelechin, outlining the biosynthetic relation between flavan-3-ols and proanthocyanidins (Fig. 1). Finally, sub-cluster A.3 included several flavan-3-ols and flavonols, together with the minor anthocyanin pigment, cyanidin hexose. Cluster B encompassed the remaining 46 secondary metabolites which were grouped into two subclusters (B.1 and B.2). Subcluster B.1 encompasses most phenolic acids (i.e. hydroxycinnamic and hydroxybenzoic acid derivatives) and the five identified terpenoid compounds. Subcluster B.2 was mainly composed of flavonoid compounds, belonging to the different flavonoid subclasses, comprising most anthocyanins including the major strawberry pigment, pelargonidin hexose.

**Correlation Analysis of Phenylpropanoids**

Strong positive correlations were observed among most proanthocyanidins and (epi)catechin both years, with correlation coefficients varying from 0.981 to 0.220 and from 0.973 to 0.210 for 2013 and 2014, respectively. Similarly, positive correlations were observed between (epi)afzelechin isomer 1 and many proanthocyanidins. Other conserved positive correlations were observed for some flavonoids, such as pelargonidin hexose with the flavanones, naringenin chalcone hexose (correlation coefficients of 0.62 and 0.74) and the two isomers of eriodictyol hexose (correlation coefficients > 0.90 for both years). Cyanidin hexose also showed strong positive correlations (> 0.9 for both harvests) with the two isomers of epicatechin glucuronide and with kaempferol-hexose isomer 1. Positive correlations were also obtained between the different derivatives of pelargonidin anthocyanins. In addition, other groups displaying repeated positive correlations in the two harvests were between hydroxycinnamic acid derivatives (ferulic, sinapic and coumaric acid derivatives) and terpenoids. The number of negative correlations between secondary metabolites was much lower than that of positive correlations. Only 103 and 45 significant negative correlations were detected in 2013 and 2014, respectively (Supplementary Table S2). Interestingly, negative correlations were observed between rutin 1 and some proanthocyanidins in both seasons, indicating a possible competition for precursors between the different branches of the flavonoid pathway. Negative correlations were also observed between hydroxycinnamic acid derivatives, such as coumaric acid derivatives, and different flavonoids, such as pelargonidin derivatives, kaempferol-hexose 2 and propelargonidins.

## Clusters of QTL for Phenylpropanoid Metabolites

Clusters of QTLs related to proanthocyanidin metabolites were detected in LG I-2, LG V-2 and LG V-3. In LG I-2, mQTLs for the flavan-3-ols catechin and (epi)afzelechin colocalized with 18 mQTLs for propelargonidins and procyanidins, suggesting the presence of a *locus* upstream of tannin condensation. The majority of those QTLs were detected only in 2013, the year where higher H^2^ values were observed, and colocalized in the same chromosomal region with *qTPC-I-2-2013*, a QTL detected the same year contributing to 17% of total polyphenol content variance.

Clusters of mQTLs for hydroxycinnamic acid derivatives were detected along the linkage map, standing out two hot spots on LG IV-2 and LG IV-3, grouping seven and ten mQTL for different phenolic acid derivatives, respectively (Fig. 4). For two isoforms of coumaric acid hexose and for cinnamic and ferulic acids hexoses, mQTLs were detected in both regions. Although the region on LG IV-2 and IV-3 contributing to the variation in hydroxycinnamic acid derivatives was detected in the upper and lower part respectively, it may be possible that homoeologous genes may control the variation of those metabolites.

For terpenoid metabolites, isolated mQTLs controlling the variation of a number of them were detected on HGs III and V, and small mQTL clusters on HGs II, IV and VII (Fig. 4). Putative stable homoeo-QTLs controlling sesquiterpenoid hexose isomer 1 were detected on LG IV-3 and IV-4, while a stable mQTL for sesquiterpenoid hexose isomer 2 was detected on LG V-3.

In HG VI, mQTLs for the three isomers of (epi)afzelechin were detected on linkage groups LG VI-1, LG VI-2 and LG VI-7, suggesting again the possibility of homoeo-QTLs. The majority of them were stable over the two years and explained a large proportion of variance. On LG VI-1, mQTLs for (epi)afzelechin collocated, among others, with a major and stable mQTL for diosmetin acetyl hexose, the only detected flavone in the ‘232’ × ‘1392’ population. On LG VI-2, mQTLs for (epi)afzelechin isomers collocated with a stable mQTL contributing to 26-36% of variation on sinapic acid hexose 2, which was also detected in 2014 on LG VI-7. mQTLs for kaempferol derivatives were detected also on HG VI, on LG VI-1, VI-3, VI-4 and LG VI-6, being one of them, *qK-phGn-9.55-VI-6*, stable and contributing to 32-34% of phenotypic variance.

**Supplementary Discussion**

The hotspot on LG IV-3 (and also on IV-2) included mQTLs controlling several hydroxycinnamic acid derivatives, some being major QTLs and stable over the two years, such as *qCouhex-5.43-IV-3*, controlling 26-34% of variance on coumaric acid hexose 2 (Fig. 4; Supplementary Table S3). Interestingly, QTLs controlling similar hydroxycinnamic acid derivatives have been detected in approximate the same location on a *F. vesca* NIL collection^1^. Urrutia and collaborators^1^ described possible candidate genes in the *F. vesca* genomic region including 3 *Fra a* genes that have been reported to be important in the control of the flavonoid pathway^2^. *Fra a* genes lay outside the QTL intervals on LG IV-2 and IV-3. However, the *LAR* gene (*FvH4_4g28110*; Fig. 1) is located within the confidence interval of QTLs on LG IV-2 and two of the characterized *4CL* genes, *FvH4-4g23640* and *FvH4-4g09340* (Fig.1), are located just outside QTL intervals on LG IV-2 and IV-3, respectively. It is well-known that 4-coumaroyl-CoA ligase is implicated in the early steps of the phenylpropanoid pathway and therefore they represent interesting candidate genes for further characterization of QTLs on LG IV-2 and IV-3.

Another cluster of QTLs was observed in LG I-2 controlling the content of different flavonoids, mainly proanthocyanidins and flavanols. Only one of the mQTL, that for (epi)catechin, was detected in both years, while the majority of mQTLs for proanthocyanidins were detected on 2013, as the QTL controlling TPC in the same region. In *F. vesca*, Urrutia et al.^1^ detected several QTLs controlling similar compounds on LG 1, also not stable over the harvests, and found a number of candidate genes in the region which could be involved in the levels of these metabolites, such as *FaFHT* or *FaCAD1* and *2*. However, these genes lay outside the hotspot region in the ‘232’ × ‘1392’ population.

References

1. Urrutia, M., Schwab, W., Hoffmann, T. & Monfort, A. Genetic dissection of the (poly)phenol profile of diploid strawberry (*Fragaria vesca*) fruits using a NIL collection. *Plant Sci.* **242**, 151–168 (2016).
2. Muñoz, C. *et al.* The strawberry fruit fra a allergen functions in flavonoid biosynthesis. *Mol. Plant* **3**, 113–124 (2010).

**Figure S1** Frequency distribution, variance analysis and correlation matrix for TPC and TEAC in 2013 and 2014.

**Table S1** Secondary metabolites profiling of '232' × '1392' F1 population for the harvest 2013. Values are relativized to the '1392' parental values.

**Table S2** Pearson pairwise correlation for secondary metabolites in the 2013 and 2014 harvests. Only significant (P<0.05) values are indicated. Metabolites in the 2014 harvests are ordered following 2013 clusters (Fig. 2)

**Table S3** QTL detected for total polyphenols (TPC), antioxidant capacity (TEAC), and polar secondary metabolites in the ‘232’ × ‘1392’ linkage map by Kruskal-Wallis (KW) and restricted multiple QTL mapping (rMQM) analysis. The position of the LOD peak (in cM), the markers used as cofactors and the 1-LOD confidence interval are indicated. The estimated mean effect (for transformed data) of the QTL (mu) associated with each of the genotypes (ac, ad, bc, bd; for the ab x cd configuration) with phase type {00} is shown. Those QTLs identified in two years are indicated in bold.

**Table S4** Relative content of metabolites with mQTLs in LG V-2-1 and LG V-2-2 in contrasting F_1_ lines with low (in green) and high (in yellow) relative content. The six lines highlighted in red and blue were selected for RNA extraction and qPCR.

**Table S5** Relative content of metabolites with mQTLs in LG V-4 in contrasting F_1_ lines with low (in green) and high (in yellow) relative content. The six lines highlighted in red and blue were selected for RNA extraction and qPCR.
