# Supplementary material for: Genetic analysis of phenylpropanoids and antioxidant capacity in strawberry fruit reveals mQTL hotspots and candidate genes
Source: Sci Rep. 2020 Nov 19;10:20197. doi: 10.1038/s41598-020-76946-x (PMC7677386; doi:10.1038/s41598-020-76946-x)

Figure S1a. Frequency distribution and analysis of variance of total polyphenol content (TPC) among the F_1 progeny. Mean of the two parents are shown in red and blue.

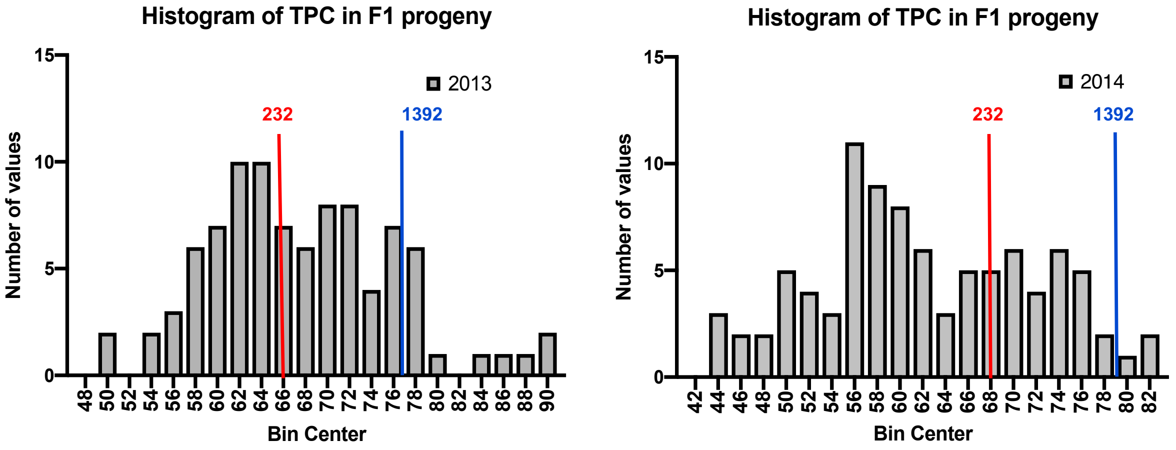

Variance analysis (ANOVA) for TPC in two years					
	**SS**	**DF**	**MS**	**F (DFn, DFd)**	**P value**
**genotype x year**	16787	91	184.5	F (91, 183) = 4.647	P<0.0001
**genotype**	26682	91	293.2	F (91, 183) = 5.575	P<0.0001
**year**	4080	1	4080	F (1, 183) = 102.8	P<0.0001
**Replicate**	9625	183	52.59	F (183, 183) = 1.325	P=0.0289
**Residual**	7264	183	39.69		

Figure S1b. Frequency distribution and analysis of variance of trolox equivalent antioxidant capacity (TEAC) among the F_1 progeny. Mean of the two parents are shown in red and blue.

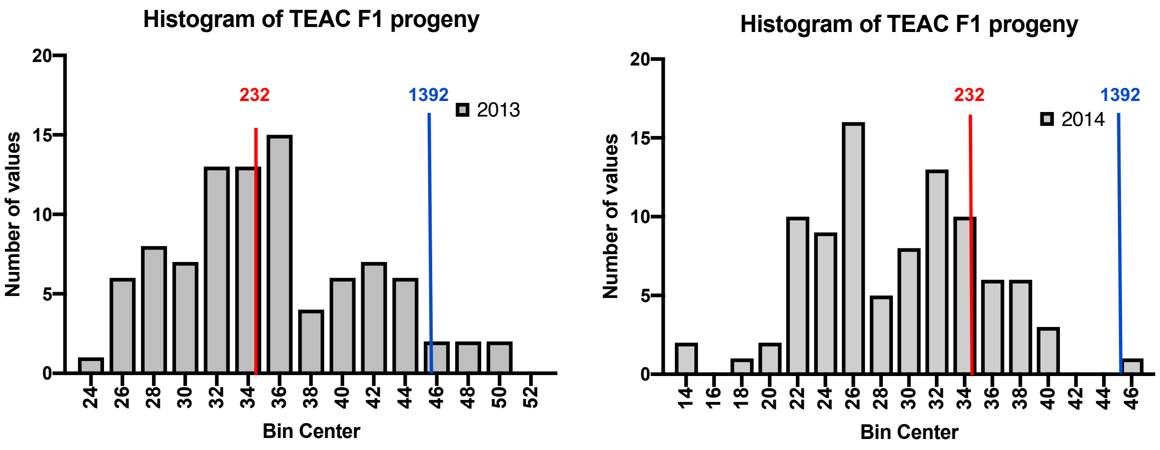

Variance analysis (ANOVA) for TEAC in two years					
	**SS**	**DF**	**MS**	**F (DFn, DFd)**	**P value**
**genotype x year**	10008	91	110	F (91, 184) = 4.456	P<0.0001
**genotype**	10499	91	115.4	F (91, 184) = 4.689	P<0.0001
**year**	5164	1	5164	F (1, 184) = 209.2	P<0.0001
**Replicate**	4528	184	24.61	F (184, 184) = 0.9970	P=0.5082
**Residual**	4542	184	24.68		

Figure S1c. Correlation matrix based on Pearson correlation analysis between TPC and TEAC in 2013 and 2014

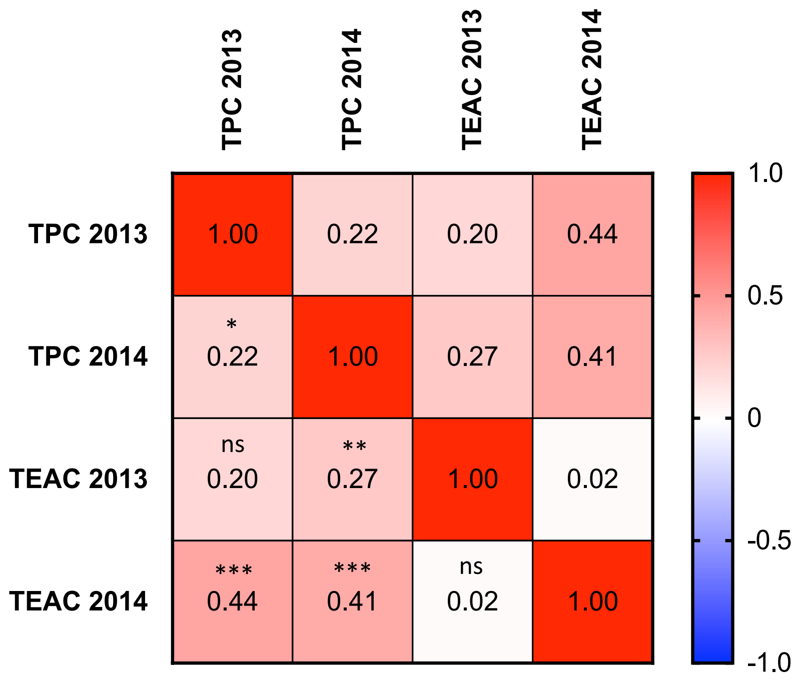

Supplement: Supplementary file 7 — Supplementary Figure. [file 41598_2020_76946_MOESM7_ESM.pdf]
